# Repopulating Decellularized Kidney Scaffolds: An Avenue for *Ex Vivo* Organ Generation

**DOI:** 10.3390/ma9030190

**Published:** 2016-03-11

**Authors:** Robert A. McKee, Rebecca A. Wingert

**Affiliations:** Department of Biological Sciences, Center for Stem Cells and Regenerative Medicine, Center for Zebrafish Research, University of Notre Dame, Notre Dame, IN 46556, USA; Robert.A.McKee.25@nd.edu

**Keywords:** bioartificial kidney, renal scaffold, extracellular matrix, matrix-cell signaling, reseeding, renal assist device, stem cell, cell therapy, regenerative medicine

## Abstract

Recent research has shown that fully developed organs can be decellularized, resulting in a complex scaffold and extracellular matrix (ECM) network capable of being populated with other cells. This work has resulted in a growing field in bioengineering focused on the isolation, characterization, and modification of organ derived acellular scaffolds and their potential to sustain and interact with new cell populations, a process termed reseeding. In this review, we cover contemporary advancements in the bioengineering of kidney scaffolds including novel work showing that reseeded donor scaffolds can be transplanted and can function in recipients using animal models. Several major areas of the field are taken into consideration, including the decellularization process, characterization of acellular and reseeded scaffolds, culture conditions, and cell sources. Finally, we discuss future avenues based on the advent of 3D bioprinting and recent developments in kidney organoid cultures as well as animal models of renal genesis. The ongoing mergers and collaborations between these fields hold the potential to produce functional kidneys that can be generated *ex vivo* and utilized for kidney transplantations in patients suffering with renal disease.

## 1. Introduction: Bioartificial Kidneys—An Alternative to Dialysis?

### 1.1. Renal Functions and the Burden of Kidney Disease

The kidney functions to filter waste from the body, recover specific metabolites, control osmoregulation, and influence hemodynamics. The kidney can become compromised if it undergoes a severe injury or a sequence of injuries over time leading to end stage renal disease (ESRD) [1]. As such, ESRD can arise from the progression of acute kidney injury (AKI), in which there is abrupt loss of renal function, or chronic kidney disease (CKD), a pathological condition in which gradual cellular changes eliminate functionality over time [2,3]. While the pathologies of AKI and a number of CKDs have been investigated in various animal models [4,5,6,7,8], there are currently only two available renal replacement treatment options for individuals with ESRD: dialysis and transplantation. Dialysis involves the use of an external filtering device to remove waste and other toxins from the blood. While this procedure can prolong the lives of individuals suffering from ESRD, it does not completely replace renal function as it only recapitulates the filtration role of the organ [9]. Transplantation on the other hand can completely replace renal function, but requires the recipient to prescribe to a life-long regiment of immunosuppressant drugs [10,11,12]. Additionally, the demand for kidneys needed for transplantation far exceeds the availably of donated organs across the globe. For example, in the United States, over 120,000 individuals currently await donation [13]. These individuals make up over 80% of people on an organ wait list in the nation as a whole. This extensive wait time is due in part to the incidence rate of ESRD and a lack of transplant quality organs [14].

### 1.2. Bioartificial Kidney Devices

In order to address these concerns, researchers have aimed to develop scaffolds upon which cells can be placed in order to provide a synthetic kidney capable of performing all facets of renal function. Work to create replacement organs has included studies using artificially created scaffolds, or patches, on which cells could be grown with transplantation occurring after sufficient cell number was achieved [15,16,17]. This approach was applied to fashion renal replacements as well, where bioartificial constructs were designed and tested to offer an alternative solution to previously existing therapies such as dialysis. The first of these synthetic kidneys was the bioartificial renal tubule assist device (RAD), designed and tested by Humes and colleagues [18]. The construct consists of a modified hemofiltration cartridge, which is seeded with renal cells, and then the cartridge is attached to a pump to allow fluid flow, either from a patient or flask containing media [19]. Initial testing using cells isolated from porcine kidneys showed the ability of these donor cells to adhere to the cartridge and maintain a differentiated state [20]. Additionally, dogs that underwent bilateral nephrectomy to create a uremic kidney environment, and were subsequently treated with a RAD, had decreased blood urea nitrogen levels compared to sham treated individuals indicating an increase in renal function [21].

Clinical studies with these artificial kidneys indicated that RAD treatment increased the renal function parameters of patients admitted to the intensive care unit (ICU); however, end point mortality at 28 days was not improved [22,23]. Over the next several years, improvements were made to RADs leading to the bioartificial renal epithelial cell system (BRECS). The BRECS design revolves around seeding cells onto coated carbon disks rather than a hollow tube and allows for cryopreservation [24]. Overall, however, several drawbacks emerged with these bioartificial kidney devices, including issues with the design that led to problems with the location of blood flow through the apparatus and clogging of pores within the units [25]. Continued work on bioartificial kidney systems encompassed testing of alternate bioreactor configurations and cellular sources [25,26]. Overall, however, a limitation to consider about these devices is that they are worn externally by the patient, thus being extracorporeal in nature. An attractive alternative would be the creation of a device that could be both effective over the long-term and capable of being housed safely within the patient. 

### 1.3. The Emergence of Techniques to Perform Cellular Seeding of Decellularized Organ Scaffolds

Within the medical field, a promising alternative to the external organ replacement devices has in fact emerged. This alternative revolves around a cell therapeutic approach using scaffolds from decellularized organs that can be repopulated with a patient’s own cells. A landmark proof of concept experiment reported in 2008 by Dr. Harold Ott and colleagues, demonstrated for the first time that an internal organ, the heart, could be decellularized resulting in an extracellular matrix (ECM) network which could be repopulated with cells that adhered to and repopulated the scaffold [27]. This seminal finding focused on the isolation and characterization of organ derived acellular scaffolds from cadavers, establishing the potential of these scaffolds to sustain and interact with new cell populations. These results launched an exciting, ongoing area of research centered on the utilization of organ scaffolds for regenerative medicine, which have opened a new vista of possibilities for the kidney field among others.

**Figure 1:**
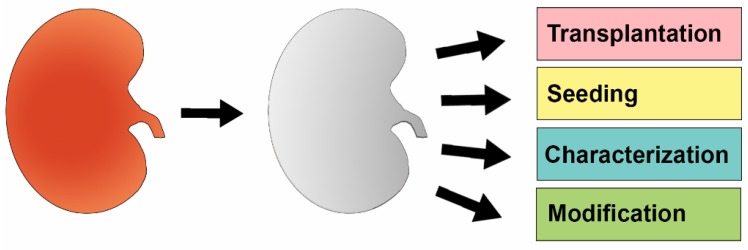
The Conceptual Basis and Application of Decellularized Kidney Scaffolds. An intact kidney (left, red) is first decellularized using agents such as detergent buffers. Once this process is complete, the resulting extracellular matrix (ECM) scaffold (middle, grey) can be manipulated in various ways, such as to be transplanted into a recipient or to undergo reseeding with new cells. Further, characterization of ECM proteins and chemical modification of the scaffold can also be performed.

In this review, we focus on the isolation, characterization, and utilization of organ scaffolds with respect to the kidney, and explore how these native networks can be used to support cellular growth and differentiation, with the penultimate goal of leading to a closer physiological recapitulation of kidney function (Figure 1).

## 2. Decellularized Kidney Scaffolds—Cadaveric Organs Supporting Life

One of the major challenges of utilizing organ-derived scaffolds is the necessity of removing all existing cellular material. From a scientific standpoint, this is critical to insure that the successive cell growth and differentiation of the seeded cells is due only to cell-matrix signals and signals between introduced cells. It is also imperative to make sure no cells remain from the original donor to avoid immunological rejection in a transplant setting. In the subsections below, we explore the methodologies that have been utilized for decellularization and the types of kidney sources and reseeding cell sources that have been experimentally studied.

### 2.1. Establishment of Basic Parameters for Renal Decellularization and Preservation of the ECM

In 2010, Nakayama, *et al.*, reported their comparative studies in which they tested different methods side-by-side for removing cells from kidneys sections obtained from rhesus monkeys [28]. Comparison of a 1% sodium dodecyl sulfate (SDS) solution and 1% Triton X-100 solution showed the SDS solution at 4° Celsius yielded an acellular organ in which the extracellular component retained the greatest degree of morphological similarity to the typical intact, healthy kidney [28]. This was based on gross observation as well as hematoxylin and eosin staining, the latter of which would become a standard in the field to determine that complete decellularization of the scaffold has been accomplished [28]. The Triton X-100 solution, while showing intact ECM, had residual cellular material after the decellularization washes [28]. Of importance was that scaffolds of SDS treated rhesus monkey kidneys contained intact ECM proteins such as collagen type I and type IV, laminin, and fibronectin [28]. Furthermore, these proteins retained their spatial location based on immunohistochemistry analyses [28]. The researchers subsequently tested whether fetal rhesus monkey kidney cells could populate age-matched decellularized kidney sections [28]. After explants of the fetal cells were layered on decellularized sections, they were cultured for 5 days and analyzed throughout this interval [28]. At 3 days, the explants fused with the scaffold, exhibiting migration into it [28]. Analysis of renal markers by immunohistochemistry revealed that the migrating cells expressed progenitor markers, including PAX2 and WT1 [28]. Taken together, this study demonstrated that decellularized renal tissues were competent to support cellular reseeding.

### 2.2. Reseeding of Decellularized Kidneys with Various Types of Stem Cells

In a contemporary study to the report of Nakayama, *et al.*, a group of researchers led by Ross, *et al.*, reported decellularization and seeding studies with whole rat kidneys that were treated with murine pluripotent embryonic stem (ES) cells [29]. In these xenograft studies, the 3D reseeding was accomplished by delivering the murine ES cells through the renal artery or ureter of rat kidneys that had been previously processed using a SDS-based protocol involving 5 days of progressive cell removal to produce acellular kidneys that were almost transparent [29]. Cell delivery through the renal artery enables cell delivery through the existing vasculature, while delivery through the ureter enables cell delivery through the existing collecting system. The donor murine ES cells were labeled with green fluorescence protein (GFP), enabling subsequent visualization of the delivery as well as imaging of cell outgrowth and migration [29]. The whole organ was incubated in growth media with either no agitation or the addition of mechanical forces through a perfusion system [29]. The donor cells were found to colonize both vascular and glomerular structures [29]. Interestingly, in a follow up to this study, the authors showed that a decellularized rat kidney scaffold that had been seeded with mouse ES cells subsequently produced new ECM, as evidenced by the presence of mouse α1α2α1 collagen IV [30]. Further, the mouse ES cells were shown to become VEGFR2 positive within two weeks post seeding, indicating their differentiation into an endothelial lineage [30]. A subsequent work reported improvement of rat kidney recellularization with mouse ES cells through the implementation of an alternative protocol [31]. Rather than decellularize with SDS over a course of 5 days, a total of 17 hours was shown to be successful, and the preservation of 3D architecture within the kidney was demonstrated using micro-computerized tomography technology [31]. Following seeding through the renal artery, the donor cells were detected in vascular and glomerular networks and occasional tubules as well [31].

In related studies, Song *et al.* used kidneys obtained from rat, porcine, and human donors, which were decellularized using a 1% SDS solution via renal artery perfusion [32]. As in previous trials, the resulting scaffolds were found to be acellular and to have an intact ECM network including physiological arrangement of glycosaminoglycans [32]. The authors then reseeded the acellular scaffolds with human umbilical venous endothelial cells (HUVECs) via the renal artery and rat neonatal kidney cells (NKCs) via the ureter in a vacuum seeding chamber, which created a pressure system that aided engraftment of the cells into the core of the scaffold. The authors found that this pressure system was necessary to allow for cell dispersion and did not cause tissue damage [32]. Importantly, their culture system employed a static culture period to allow cell adhesion to occur, followed by perfusion culture to supply oxygen and nutrients likely promoted efficient engraftment of HUVECs and NKC populations. After engraftment, the authors used an undisclosed combination of growth factors to drive NKC differentiation. Additional analysis of these reseeded scaffolds showed that the seeded cells homed to their physiologically appropriate niches with HUVECs reconstituting endothelial vessels and capillaries while NKCs formed tubule components. Furthermore, some of the NKC derived tubular structures expressed Na+/K+-ATPase, likening them to proximal tubular epithelium, while other derivatives exhibited E-cadherin expression, thus resembling the distal tubules, collecting ducts, and transitional epithelium in the renal pelvis [32]. Importantly, electron microscopy allowed for the observation of glomeruli, in which podocytes engrafted and formed foot processes [32].

Notably, the study by Song *et al.* was among the first to include transplantation experiments in rats, in which reseeded scaffolds where attached to the circulation of left-nephrectomized individuals [32]. These orthotopically transplanted bioartificial scaffolds were found to restore glucose and electrolyte reabsorption by almost 50% and produce urine; however, similar to the RADs, some of the specific parameters were less efficient than decellularized scaffolds, likely due to a semi-immature renal environment especially in the glomeruli [32].

Similar experiments were performed by Guan *et al.*, in which rat scaffolds were seeded with murine embryonic cells via the renal artery and the ureter as well [33]. Orthotopic transplantation of reseeded scaffolds into rats allowed for initial blood flow and no observable signs of rejection were observed; however, by two weeks post implantation, the renal artery and vein had thrombi blocking sufficient blood flow [33]. This is likely due to an absence of the correct cell type in the vasculature, which was provided by the HUVECs in the study by Song *et al.*, as well as the inclusion of growth factors by the latter group to aid in differentiation of the NKCs [32].

### 2.3. Further Optimization of Reseeding the Decellularized Kidney

Taken together, these experiments established the capacity of kidneys to be harvested from animals and cadavers and have their cellular components removed, yet still retain sufficient nascent elements to support the growth, migration, and differentiation of newly introduced cell populations. The studies involving transplantation of the scaffolds used stem cells of embryonic or fetal origin to seed the cleared extracellular matrices. In a clinical setting, it would be more advantageous if these scaffolds could be thoroughly populated—the identification of alternative cell sources may provide one avenue to improve this aspect. For example, induced pluripotent stem cell (iPS) derivatives or cells from an adult organ might be more effective in engraftment than the rates observed in the Nakayama and Ross studies.

In 2014, experiments were performed that partially addressed this issue. In a study by Yu *et al.* kidneys were isolated from adult rats and decellularized with washes of solutions containing both Triton X-100 and SDS [34]. Subsequent histological staining techniques and vascular corrosion casting showed this decellularization technique effectively removed cells while leaving an intact ECM. Decellularized scaffolds where then cut into thirds and sutured to host kidneys that had undergone a 1/3rd nephrectomy. Analysis showed that the host tissue directly adjacent to the implanted scaffold to become infiltrated with inflammatory and renal parenchymal cells which took on glomerular and tubular morphologies, however, this was replaced by scar tissue by eight weeks post operation.

Another issue of clinical relevance is the respective size of the organ being used. Studies have been performed on various kidneys including porcine, in part due to their relatively comparable size to human kidneys (the latter typically measures 10–12 cm long) [35,36,37,38]. The ability to decellularize and reseed kidneys of this scale to the same extent as the smaller rat kidney (typically 1–2 cm long) is a critical aspect; of further note is that porcine reseeded scaffolds have also been implanted into recipients [35,36,37,38]. Importantly, in further work with human kidneys, immunostaining of the acellular ECM revealed that it was absent of two HLA antigens, HLA-ABC and HLA-DR which represent the human major histocompatibility complex class I and II respectively, and that are involved in the immunogenic graft rejection response [39].

Together, the above studies document the ability of the renal extracellular matrix to undergo decellularization while remaining intact, providing a platform for subsequent reseeding and production of a semi-functional kidney; however, a lack of efficient differentiation into the many normally occurring renal cell types hinders their efficacy in clinical settings at the present time. Additionally, the use of human-derived ES cells in clinical settings is controversial and adaptation of human iPS cell derivatives into the acellular scaffolds would allow for wider acceptance of the technology, though establishing the safety of reprogramming cell sources will be essential. It is also known that the differentiation processes of iPS cells are highly dependent upon and sensitive to growth factors and cytokines [40]. Therefore, in order to more efficiently reseed acellular scaffolds, a greater understanding of the specific signaling pathways occurring between the renal ECM and reseeded cells is likely to be crucial. Unravelling these signaling cues may also help guide the differentiation of induced pluripotent stem cells.

## 3. Acellular Environment—Environment Directed Differentiation

### 3.1. Assessment of ECM Reseeding in Different Kidney Regions: Mouse and Porcine Xenograft Studies

Initial characterizations of the acellular kidney scaffolds showed that the ECM remained intact after decellularization with detergent solutions; however, the compositions between particular regions of ECM were not specifically scrutinized. An interesting study by O’Neill *et al.* isolated the ECM from the cortex, medulla, and papilla regions of porcine kidneys [41]. Collagen, sulfated glycosaminoglycans (sGAGs), and fibronectin in the ECM were assessed across the different kidney regions [41]. Notably, sGAG composition varied, with lower concentrations located in the cortex [41]. Mouse kidney cells from the papilla region were grown on hydrogels or decellularized scaffolds, or exposed to media containing solubilized ECM from the cortex, medulla, or papilla regions or the whole kidney [41]. Rather than the physical state of the ECM affecting growth and differentiation of the mouse papilla cells, it seemed that differences arose from the region the matrix was isolated from, such that the renal papilla ECM was associated with reduced cell proliferation and increasing metabolism [41]. The researchers also sought to assess whether exogenous (non-kidney) stem cells would respond differently in terms of their growth on ECM from different kidney regions. Interestingly, when they used mouse bone-marrow derived mesenchymal stem cells (MSCs) as an exogenous test case, in which the MSCs were exposed to ECM from the aforementioned regions in an analogous manner, no differential effects on either growth or metabolism were observed [41]. However, the morphology differed between mouse papilla cells when cultured on ECM sheets from the different kidney regions [41]. The cortex seeded mouse papilla cells aggregated and took on a star-like morphology [41]. In comparison, mouse papilla cells cultured on medulla ECM sheets elongated and began to form tubular structures [41]. Further, mouse papilla cells cultured on papilla derived ECM sheets appeared as clusters of cells with a rounded morphology [41]. These findings suggest that the overall architectural differences between regions of the ECM can affect cell morphology [41]. Additionally, it is possible that small molecules left behind after decellularization may have an impact on cellular dynamics. This data, together with additional experiments in the study showing that bladder and heart ECM derivatives did not affect mouse papilla cells differentiation, proliferation or metabolism, suggests that the response to ECM cues is cell-type specific [41].

### 3.2. Assessments of ECM Composition and Xenograft Studies with Mammalian Kidneys and Human Stem Cells

The outcomes of other xenograft studies, this time with decellularized rat kidneys and human embryonic stem (HES) cells, were reported by Finesilver, *et al.*, in 2014. In this experimental paradigm, the HES cells were cultured on so-called kidney-derived microscaffolds (KMSs). The KMSs were organ fragments prepared by obtained by collecting rat kidney sections (300 µm in thickness) and then processing these tissue samples through a decellularization procedure involving a series of short washes in distilled, millipore-filtered water, followed by short washes in 0.5% Triton X-100 and an overnight incubation in saline [42]. This 2-dimensional approach is similar to the section-based study performed by Nakayama, *et al.*, where they cultured fetal monkey kidney cells on tissue sections of decellularized rhesus monkey kidney specimens, but the KMS were not adhered to a slide [28]. The KMS acellular fragments were shown to be capable of supporting engraftment of HES cells and human proximal tubule (HK-2) cells, as well as Madin-Darby canine kidney epithelial cells (MDCK) [42]. Furthermore, Finesilver, *et al.* found that after two weeks in culture, the HES cells that had engrafted in KMSs showed an increase in the expression of several kidney genes including the glomerulus-associated gene *NPHS-1,* and the tubule-specific genes *AQP-1* and *SLC2A2*, as well as a shift away from stem cell morphology [42].

One notable contribution made by Finesilver, *et al.* was that they performed mass-spectrometry on their KMS samples, which they solubilized to determine the identity of structural proteins and other peptides present in the renal ECM [42]. This information is valuable for future studies because ECM protein composition has a dramatic impact the physical and biomechanical structure of tissues. It was noted that many differences exist in the makeup of ECM derived from the kidney compared to lung and bladder, especially with regard to collagen composition. Knowledge about these characteristics will be useful for understanding the molecular basis of how seeded cells recognize their environment and behave during the recellularization process, which may reveal ways to improve this process for various organs. 

Another xenograft study utilized the same line of HES cells, but cultured them on 8 mm acellular rhesus monkey renal biopsies [43]. In this context, the HES cells began to express renal tubule markers by two days with a continual increase in the level and number of genes expressed by eight days based on qPCR analysis [43]. Additionally, proteomic analysis between renal and lung acellular scaffolds indicated that the renal ECM contained over 200 proteins not present in the lung, while 110 shared proteins were identified in both samples [43]. These unique proteins included keratins, antimicrobials, and growth factors including TGF-β and EGF-7 [43]. To ensure that differences in differentiation potential were due to the presence of proteins, the authors assessed mRNA levels in the scaffolds and found a lack of mRNA in the biopsies after decellularization [43].

Taken together, the studies discussed in this section show that the renal ECM contains specific protein and glycan identities compared to other organs with an even greater refinement of architecture and ECM composition in sub-regions. Furthermore, the ECM composition remains relatively intact after undergoing decellularization procedures. The importance of the unique ECM structure lies in its ability to dictate the microenvironment in a meaningful way to influence cellular differentiation. Continuing to better understand of the nascent molecular components of the renal ECM, and the dynamics of ECM within the intact kidney during health and disease states, will likely be essential in recapitulating complete renal function in reseeded scaffolds.

## 4. Summary and Future Directions for the Nephrology Field

Within the past decade, multiple research groups have explored the feasibility of producing acellular renal scaffolds harvested from animals or human cadavers. Decellularization with detergent solutions, most notably SDS, yields scaffolds absent of donor cells which can then be populated, *i.e.* reseeded, with stem cells which will undergo differentiation based on cell-matrix signaling via molecular cues embedded within the ECM as well as the architectural structure of the scaffold itself. Such studies built off attempts to create bioartificial devices, with the goal of reducing transplantation wait time and providing better therapies for ESRD then the current options for renal replacement therapy. While these attempts have yielded both smaller (rat) and larger (porcine) reseeded scaffolds for transplantation, the field can benefit from continued advancements in other areas of stem cell biology.

Recent investigations have shown that ES cells and iPS cells can be stimulated to differentiate into multiple renal cell types which will interact with each other to form semi-organized structures containing both tubule and glomerular components, termed organoids [44,45]. These organoids have been shown to respond to injury in a similar manner to whole *in vivo* organs [46]. A recent *in vitro* study described a method by which human adult proximal tubule cells (HK2) could be reprogrammed into an embryonic state [47]. Applying the knowledge gained from these studies, namely the identity of the growth factors and cytokines utilized to reprogram these cell types into different states of potency, could increase the capacity of cells to seed into acellular matrices and differentiate more effectively. For example, these molecules could easily be added to media before perfusion in *in vitro* bioreactor systems. Related to such pursuits, the continued study of renal genesis in animal models, both during development and in the context of kidney regeneration, is likely to provide valuable insights into the complex pathways of recellularization.

Another exciting avenue for the field comes with the advent of 3D printing which has been used to create cell scaffolds for other tissues [48,49]. This technology has been adapted most recently for so-called “bioprinting,” in which biomaterials such as collagen microfibers and ECM components are built in precise spatial positions along with the direct deposition of various cells or cellular aggregates [48,49,50]. In these studies, complex fabrications have been assembled that include vasculature, ECM and multiple cell types, ranging from bone mesenchymal stem cells to chrondocytes [50,51,52,53]. At present, the success of tissue reconstruction using bioprinting is reliant on the concept that the cells will have the capacity to self-organize and create typical functional arrangements when placed within the context of an appropriate microenvironment context. For example, a system of 3D printed biomaterials has been utilized to stimulate nerve regeneration with spatially controlled axonal guidance, in which path-specific biochemical cues were deposited along the scaffold [54]. Given these advances, detailed characterization of the decellularized renal scaffolds could lend to multi-dimensional reconstruction through bioprinting, rather than obtaining these scaffolds from animal or human cadavers. If this prospect is realized, it would have the ability to eliminate the waiting list for transplantation.

While enormous progress has been made with bioartificial kidneys and reseeding acellular scaffolds, there still remain critical questions for the field to address. First, it is unknown how conserved the ECM is across different species. This has important implications for the transition from animal models into human samples and clinical settings. Furthermore, there is a gap in the detailed information available on how the ECM is altered in disease states such as AKI and polycystic kidney disease (PKD). Elucidation of the renal ECM composition in such aberrant states, by studying decellularized kidneys from such patients, could reveal powerful insights about the pathology and possible treatments for these diseases. Finally, the signaling cues allowing reseeded cells to home to their proper niches remains uninvestigated. In answering these questions, insights will be gained into how the kidney develops properly *in vivo*, and how we can recapitulate this development using isolated extracellular matrix *in vitro*, for subsequent transplantation into critically ill patients.

## Abbreviations

The following abbreviations are used in this manuscript:

AKI: acute kidney injury
BRECS: bioartifical renal epithelial cell system
CKD: chronic kidney disease
ECM: extracellular matrix
ES: embryonic stem
ESRD: end stage renal disease
GFP: green fluorescent protein
HES: human embryonic stem
HUVECs: human umbilical venous endothelial cells
ICU: intensive care unit
iPS: induced pluripotent stem
KMS: kidney-derived microscaffolds
MSC: mesenchymal stem cell
NKCs: neonatal kidney cells
PKD: polycystic kidney disease
RAD: renal assist device
SDS: sodium dodecyl sulfate
sGAG: sulfated glycosaminoglycan
